# Insect Colonisation and the Decomposition Process in Aerated versus Watertight Burial Systems

**DOI:** 10.3390/insects14060566

**Published:** 2023-06-19

**Authors:** Francesco Defilippo, Martina Munari, Annalisa Grisendi, Rosa Maria Gaudio, Mario D’Incau, Antonio Lavazza, Silva Rubini

**Affiliations:** 1 Istituto Zooprofilattico Sperimentale della Lombardia e dell‘Emilia-Romagnia (IZSLER), Via Bianchi 7/9, 25124 Brescia, Italy; martina.munari@izsler.it (M.M.); annalisa.grisendi@izsler.it (A.G.); mario.dincau@izsler.it (M.D.);; 2 Section of Legal Medicine and LTTA Centre, Department of Translational Medicine, University of Ferrara, 44121 Ferrara, Italy; atf@unife.it

**Keywords:** burial system, *Megaselia scalaris*, aerated tomb/mound, *Hydrotaea capensis*

## Abstract

**Simple Summary:**

Aerated burial systems involve slow air circulation within a mound, caused naturally or artificially. The weak airflow allows bacteria to work in an aerobic environment, accelerating the natural skeletonisation process. The main objective of this study was to document the decomposition level of an animal carcass in an aerated and watertight niche in a special epigean loculus. Pig carcasses (weighing approximately 70 kg each) were used as an experimental model for testing differences between the two systems. Each carcass was removed from the niches at predefined intervals, and the degree of decomposition and the presence of macrofauna were verified. The study examined if insects could colonise carcasses in the two burial systems to determine which fauna were present during the sampling dates. The possible existence of bacterial colonies was also assessed after five years. Our experiments showed that aerated burials facilitate skeletonisation similarly to watertight burial methods. Therefore, aerated burials are a viable alternative to watertight burials in terms of reducing both decomposition time and the costs of urban cemetery planning. It is also an alternative to interment in cemeteries where the soil cannot properly skeletonise. Aerated entombment applies to both hypogeum and epigean solutions.

**Abstract:**

In recent years, burial systems have covered increasingly higher costs due to the pollution caused by decomposition products. These products are understood as chemicals and microorganisms in the surrounding soil and groundwater and represent a topical issue. The purpose of this research was to ascertain the extent of decomposition when pig carcasses are buried in two different burial systems (“aerated” vs. “watertight”) and catalogue the arthropods associated with burials at different time-points of removal from niches (after 6, 12, 24, 36, and 60 months). Thirteen taxa were collected in aerated niches, whereas five were collected in watertight niches. The initial access or exclusion of insect colonisers affected overall functional activity. Two Diptera species, *Hydrotaea capensis* and *Megaselia scalaris*, were the most abundant, supporting the hypothesis that insects can colonise carcasses in aerated burial systems. Furthermore, some species of bacteria have been documented as facilitators of the initial decomposition process of the carcass. Most bacterial colonies develop only in aerated niches. The trial showed that the first enzymatic–bacterial and insect actions helped promote the process of cadaveric decomposition and later skeletonisation, mainly when associated with aeration modes of the tomb/mound. The results obtained provide essential information on the process of human decomposition and taphonomy in cemeteries. Moreover, these data could benefit forensic science by adding information on insect colonisation and body modification in medico-legal investigations concerning the post-mortem interval in exhumed bodies and illegal burials.

## 1. Introduction

In recent years, burial systems have become increasingly expensive, especially in urban planning, where there is a shortage of space for cemeteries in populated areas. However, they also harmfully impact the environment and health. The processing of corpses, from autopsy to burial or cremation of mortal remains in a columbarium, generates several potential contaminants that can disperse into the environment [1].

Buried corpses can be sources of various chemical and microbiological contaminants. If the cemetery is located on vulnerable grounds or the soil reaches its depurative limit, these contaminants could reach the groundwater. In this case, the main risk to public health is the spread of waterborne diseases by direct or indirect contact with contaminated water or pathogens’ vectors [2].

The global population is nearing eight billion, limiting the amount of land available for human burials, especially in small and densely populated countries.

The lack of burial spaces in cemeteries is now a widespread problem.

The situation in Italy is evident through a simple search. Dozens of municipal administrations, mainly medium to large urban centres, face a real ‘space emergency’. In its online publication, the BCC devoted an interesting article to the global crisis faced by numerous cemeteries, including the lagoon cemetery in Venice [3]. Coffins used for burial in Italy are fabricated from wood, and metal coffins are added for watertight entombment.

The high number of ‘mortal remains’ found after stagnant burial times is a topical issue. The stagnant burials are those that are watertight, that is, impermeable to liquids and gases. It strongly discourages, slows down, and sometimes stops the corpse’s decomposition, and has several long-lasting harmful effects on the environment. In-ground burials also exhibit delays in the decomposition, even if in a ratio of at least 1:5 for watertight entombments, especially if both the soil and groundwater levels are unsuitable. Wood and metal fragments from coffins and caskets remain in the ground, releasing harmful chemicals through paints, additives, preservatives, and alloys. Cemetery pollution can cause changes in the microbiological components of soil and groundwater by increasing the concentration of heavy metals and toxic organic pollutants when additives such as antidepressants are used [4,5]. Although human cremation is also an increasingly common practice, there have been few studies on the potential risks of crematorium emissions compared to the effects of more hazardous compounds (PCDD/F and mercury) and lesser-known compounds (NOx, CO, SO_2_, PAHs, etc.) [6].

Therefore, the most environmentally sustainable choice is the one that allows the body to decompose and skeletonise rapidly.

Green burial is designed to have a minimal environmental impact and conserve natural resources. Therefore, they should include the following:

- No chemical embalming, which keeps pollutants out of the ground;
- Headstones that are biodegradable or smaller, less intrusive grave markers;
- Coffins that are made of biodegradable materials such as cardboard or wood. Materials such as finished lacquered wood and metal rails are not used in their construction;
- A shroud that is non-bleached, undyed, and made of natural fibre is used to cover a body;
- A choice of a grave that helps speed up the decomposition of human remains.

The decomposition process is influenced by numerous intrinsic and extrinsic factors, including humidity, burial type, or funerary practice chosen (inhumation or cremation), access to necrophagous insects (insects and arthropods), and scavenger activity. Fungi and bacteria can facilitate the initial decomposition processes in carcasses [7,8].

Primary insect colonisers utilise carcasses as a nutritional resource or a mating and oviposition site. Subsequent larval development may disrupt established microbial communities through direct or indirect competitive interactions on the carcass [9]. Insects arriving to colonise carcass introduce their exogenous microbial community [10], such as *Musca domestica* (Diptera: Muscidae), which carries over 100 pathogenic microbes. These microbial communities may influence the insect community. For example, putrescine is a well-known volatile compound associated with decomposing remains [11]. It attracts blow flies but repels male carcass beetles (Coleoptera: Silphidae) [12]. Putrescine is also a molecule required for swarming behaviour in bacteria such as Proteus mirabilis, commonly associated with blow flies [12].

Among these influencing factors, temperature plays an essential role in the functioning of aerated burials with natural ventilation [13].

In search of innovative burial solutions, a new type of corpse arrangement called ‘aerated burial’ may represent a new green solution. Aerated burials allow a slow circulation of air, natural or artificial, inside the niches of a grave. Due to temperature variations between day and night and solar radiation outside the tomb, this process relies on weak ventilation to expand and reduce the volume of gases inside the grave. In addition, the weak airflow allows bacteria to work in an aerobic environment, accelerating the natural skeletonisation process [14].

In aerated graves, zinc caskets and impermeable internal linings forbid air from reaching the corpse and facilitate cadaveric fluid evacuation. The latter is collected and treated differently depending on the methods used, usually with a collection tank under the coffin containing biodegradable absorbents. In addition, temporary liquid collection solutions are used inside the coffin to ensure its permeability in the lower part.

The mechanism that promotes skeletonisation is the combination of the following elements.

- Oxygen availability over time for determining aerobic conditions inside the mound that accelerate decomposition processes, which would otherwise occur mainly in an anaerobic environment.
- The evacuation of cadaveric fluids from inside the coffin, thus eliminating the stagnation of the part of the corpse in contact with a considerable amount of cadaveric liquids and thus reducing the phenomena that are contrary to decomposition [9].

In Europe, the first countries to legalise the use of aerated graves in cemeteries were France, with the standard NF P 98-049:1994 [15], and Spain, with Decree 2263/1974 (20 July 2020) and autonomous regulations [16]. Moreover, Emilia Romagna (Northern Italy), where this study was conducted, has been the only Italian region to legalise aerated graves since 2006 [14,17].

In this study, we aimed to ascertain the decomposition degree of pig carcasses buried in two different burial systems and catalogue arthropods in five different series of exhumations (after 6, 12, 24, 36, and 60 months). We discussed the burial environment’s effect on the degree of decomposition and the implications for various arthropod taxa. In addition, the insect colonisation and arrival time on buried pig carcasses were observed, establishing a preliminary dataset on the insect succession of buried carcasses in temperate climates and urban environments.

According to the EUROPEAN STANDARD EVS EN 15017 Funeral Services—Requirement [18], Supplementary File S1 contains a glossary of terms features in this paper.

## 2. Materials and Methods

### 2.1. Study Site and Animal Models

Our study was conducted at a site adjacent to Mizzana’s Cemetery (Ferrara) (44°50′46″ N; 11°34′39″ E) at an elevation of 11 m above sea level for 60 months beginning in November 2016. The burial site was approximately 50 m off a gravel road without vehicles or people passing. The vegetation primarily consisted of shrubs and grasses typical to the lowlands of Northern Italy with juvenile trees of *Robinia pseudoacacia* (Fabales: Fabaceae), *Quercus ilex* (Fagales: Fagaceae), and *Acer campestre* (Sapindales: Aceraceae).

**Figure 1:**
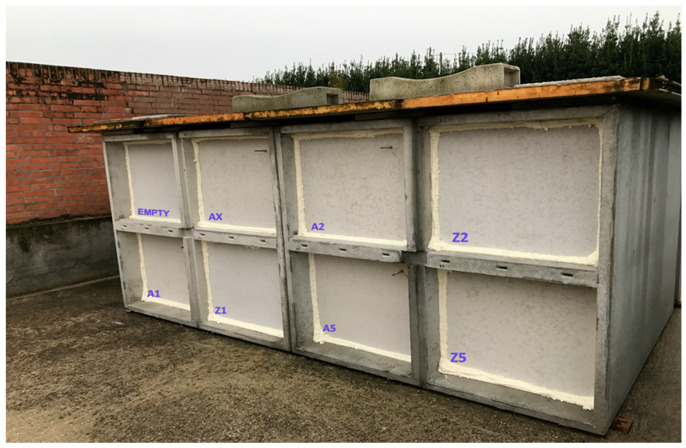
Structure of different burial niches (A = Aerated niche; Z = Watertight niche) (dimensions 230 × 80 × 80 cm).

Two floors of eight precast cement niches sealed with polyurethane-based sealing foam were used (dimensions 230 × 80 × 80 cm) (Figure 1).

Since Italian legislation prohibits using human cadavers for experimental purposes, we used pigs, considered the best proxy for human bodies for taphonomic studies [19]. Previous research recommended using pig models since pig carcasses’ decomposition pattern is comparable to humans. In addition, they contain similar microbial biomass to humans in size and composition [20]. Seven domestic pigs (*Sus scrofa* L.) weighing 70 kg were obtained from a commercial piggery in Ferrara (Northern Italy). Immediately after, the pigs were killed by stunning and according to current Italian legislation, they were eviscerated and placed in black plastic trash bags to prevent insect contamination. Then, each pig carcass was individually placed in a small pinewood coffin and sealed inside a niche to replicate entombment. Three carcasses were buried in conditions resembling a watertight tomb. Another three carcasses were buried in conditions resembling an aerated tomb. One more carcass under aerated conditions (AX) was added to be opened after six months to check for any excessive degradation of the coffin and thus modify the experimental plan by reducing the observation time. Finally, an empty niche was used to evaluate the possible degradation of sealants or cement in the absence of the carcass.

Rainfall and ambient temperature were recorded daily during the five years by the closest weather station, sited in Ferrara at about 5 km (11.621138°, 44.832498°). Both data sets referred to station Ferrara Urbana, the closest to the experimental site (website accessed on 7 August 2020). Each burial was exposed to the same ambient temperature fluctuations. The proximity of the burial sites ensured that each received approximately equal amounts of direct sunlight throughout the day. None of the surrounding foliage was sufficient in height to throw shade on the burial, and shadows from buildings were also not an issue.

### 2.2. Procedures of Exhumed

One pig carcass, originally entombed in an aerated environment set of remains (AX), was exhumed from the niche after six months. All the other pig carcasses were exhumed from niches, as in Table 1. The AX niche was used to check for excessive coffin degradation and thus modify the experimental plan by reducing observation time. Arthropods were collected manually from the external burial and the carcasses at the time of exhumation removal from the niche and placed in glass vials containing 70% ethanol for preservation. All insects and their fragments were initially morphologically identified using specific keys [21,22]. Special marquees were set up near the niches to avoid insect colonisation during exhumation operations. The study aimed to provide more reliable criteria for determining insect colonisation capacity and niche integrity by avoiding openings as much as possible during the experiment.

Each morphologically identified specimen was stored in ethanol for molecular species confirmation and further processing. After five years of experimentation, microbiological samplings were performed by swabbing different body regions, including the mouth, head, throat, thoracic skin, abdominal skin, hind limb, and hypostatic side. Furthermore, two swabs were conducted on the liquid present on two wooden crates, namely, external wet and external dry residue.

### 2.3. Molecular Insects’ Identification

After morphological identification, some specimens were individually processed for molecular analyses. DNA barcoding, the sequence variation analysis of 59 regions in the mitochondrial cytochrome c oxidase I (COI) gene, provides an efficient method for identifying species in a wide range of animal taxa. Before extraction, samples were washed three times with PBS to remove any contaminants, putrefaction products, or fragments of other insects. Then, following the manufacturer’s instructions, DNA was extracted from insects using Quiagen^©^ Biosprint one-for-all Vet-Kit (QUIAGEN, Milano, Italy) to amplify the target 658-bp fragment of mitochondrial cytochrome c oxidase (COI gene) using forward primer LCO 1490 (5′-GGTCAACAAATCATAAAGATATTGG-3′) and reverse primer HCO 2198 (5′-TAAACTTCAGGGTGACCAAAAAATCA-3′) [23]. DNA was sequenced on a MiSeq Instrument (Illumina Inc., San Diego, CA, USA), and the sequences were utilised for virus identification by BLAST analysis against the GenBank (GB) database (https://blast.ncbi.nlm.nih.gov/Blast.cgi, accessed on 27 February 2023).

### 2.4. Microbial Community Sampling

Microbial sampling was conducted on a surface of 10 cm^2^ with a swab and placed in a sterile tube with saline. After that, swabs were plated on selective and non-selective solid media: Blood Agar Medium, MacConkey Agar, Sabouraud Dextrose Agar, and Brain Heart Infusion Broth (BHI). They were incubated for 24 h at 37 °C and 25 °C for Sabouraud Agar plates. Gram staining with catalase and oxidase was performed on isolated bacterial colonies. Single colonies were used for mass spectra analysis in a Vitek^®^ MS System (Biomerieux, Marcy l’Etoile, France). They were picked fresh from agar plates and transferred to steel target plates, followed by adding 1 μL of α-cyano-4-hydroxycinnamic acid in a saturated solution with 25% acetonitrile and 25% ethanol. Colonies were analysed in duplicate for each identification event. Two different identification events were prepared using various colonies from the same isolate. The Vitek^®^ MS IVD database software compared the obtained spectra with reference database spectra and expressed the resulting similarity value as a confidence value. The highest score between the two identification events was used for identification purposes. External calibration was performed with a reference bacterial strain (*Escherichia coli* ATCC 8739) suggested by Vitek^®^ MS.

## 3. Results

Rainfall and ambient temperature are shown in Table 2 and Table 3. Seasonality is typical of the Mediterranean area, with hot and dry summers and mild and rainy winters. Indeed, mean daily temperatures fluctuate between 2.1 °C in Winter and 26.6 °C in Summer, and rainfall is usually most abundant in Spring and Autumn (Table 2 and Table 3).

**Figure 2:**
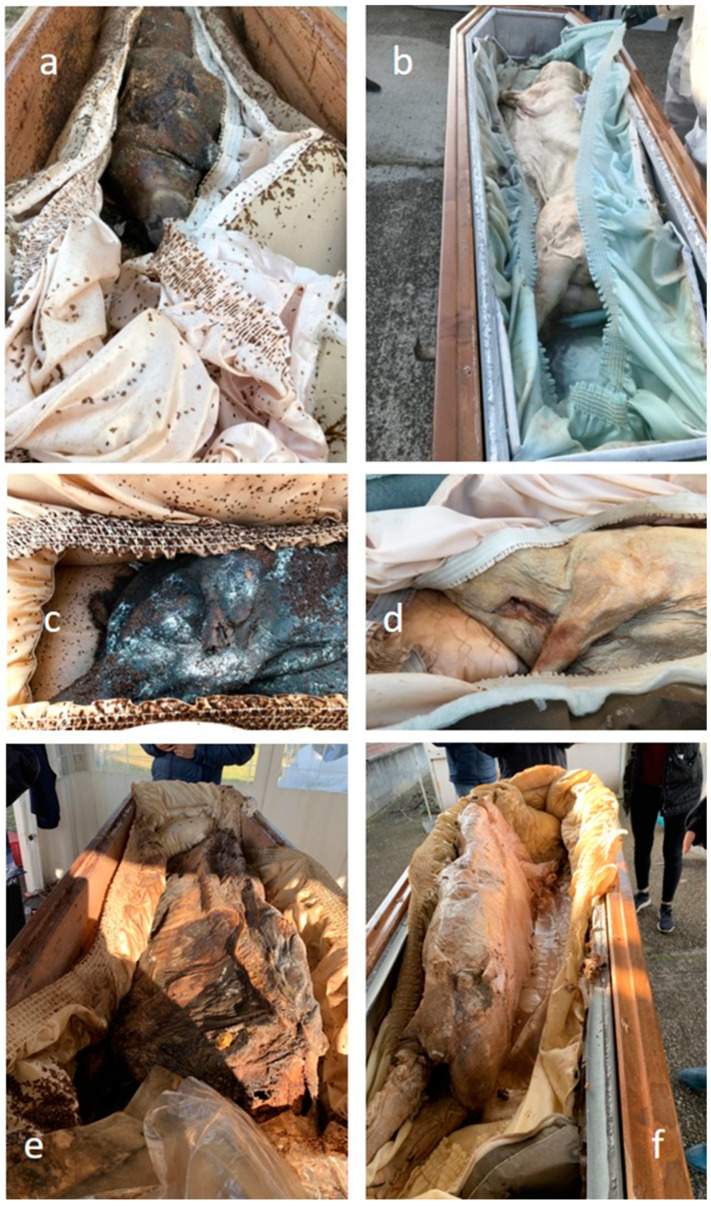
Different decomposition in Aerated (**a**,**c**,**e**) and Watertight burial systems (**b**,**d**,**f**) after 12–24 and 60 months.

The decay rate of the carcasses in watertight niches (Z) was much slower than in aerated niches (A). When the niches were opened, coffins in A had cracks, whereas coffins in Z were intact. The aerated niche (A) samples reached the drying stage after 12 months. They presented themselves in the skeletonisation phase, especially in the legs, with blackish skin of hard consistency. The epistatic areas were the most affected, while the hypostatic areas sometimes had continuity loss, and others were in a mummification state. Samples from watertight niches (Z) preserved the humidity of the soft tissues. They showed the progressive, transformative phase of saponification with classic adipocere appearances, including hard, irremovable saline granules despite strong traction. The saline granules are more represented in the epistatic areas, whereas the hypostatic regions were more affected by the colliquative stage (Figure 2). Contact with air may have affected the decomposition process and the integrity of the coffins.

### 3.1. Insect Species Taxonomic and Genetic Identification

The first signs of an insect’s colonisation showed 12 months after starting the experiment. Carcass A1 appeared in the dry stage, and only the pupal remains of *Megaselia scalaris* were collected. Carcass Z1 appeared in the adipocere stage, but no arthropods were detected or collected during inspections.

After 24 months, the most abundant taxa of pupal remains collected from Carcass A were *Megaselia scalaris* and *Hydrotaea capensis*. No arthropods were detected or collected during inspections of Z.

A1 and Z1 carcasses were exhumed after 36 months to evaluate the effects of decay and associated arthropod fauna. Three taxa of pupal remains were collected during daily inspections: *Megaselia scalaris*, *Hydrotaea capensis*, and *Monopis imella*. In Z1, the first colonisation signs occurred with the pupal remains of *M. scalaris*.

The most significant number of taxa were sampled during the final exhumation (after 60 months) (Table 4). Thirteen taxa were collected from aerated niches, whereas five were collected from watertight niches. The number of sampled insects from each burial system is reported in Supplementary File S1 and Table S2.

The high number of specimens of *H. capensis* and *M. scalaris*, termed second-wave colonisers, is consistent with long-buried carcasses. No arthropods of first-wave colonisers, such as blow flies and flesh flies, were also detected or collected.

Concerning Coleoptera, there was a noticeable variation in species on carcass in Aerated and Watertight system. In total, we identified two beetle species (*Aleochara* sp. and *Anthrenus verbasci*) in Watertight niches and seven in aerated niches. In aerated niches, after 60 months, exuviae of larvae (*Anthrenus verbasci*) and adults (*Dermestes maculatus*) were the most abundant beetle species.

DNA barcoding, the sequence variation analysis of 59 regions in the mitochondrial cytochrome c oxidase I (COI) gene, provides an efficient method for identifying species in a wide range of animal taxa. Therefore, data in GenBank were compared with the obtained COI sequences to confirm morphological identification. The results are shown in Table 5. The COI sequences of insects obtained in this study are reported in Supplementary File S1 and Table S2.

### 3.2. Microbial Community

Yeasts and moulds were not detected despite the evident presence of fungal growth on carcasses.

Bacterial growth produced poor results. The bacterial colonies only developed in aerated niches and Z1 (watertight niche) because the latter was opened twice during the trial. Table 6 shows species of isolated bacteria.

## 4. Discussion

In this study, inhumation, notoriously associated with water and fluid drainage, insect decomposers, and oxygen availability, was confirmed as the environment characterised by the fastest decomposition with complete skeletonisation of the carcasses [24,25]. The decomposition process in aerated niches leads to mummification after 12 months. Indeed, the presence of air, an essential and defining feature of aerated tombs, promoted both the efficient evaporation of fluids and the colonisation by diverse bacterial species, aerobic and anaerobic, which promote the fast decomposition of organic matter, in accordance with previous studies [25].

Entombment in watertight niches leads to the pervasive formation of adipocere. Contrary to in-ground burials, namely, inhumation and aerated niches, watertight niches feature a double coffin (wood and metal) and act as closed systems with no contact with the external environment. They create a humid microclimate inside the metal coffin that causes slow weight loss and decomposition in the absence of evaporation.

We found that overall functional activity was affected by insect colonisers’ initial access or exclusion. Most insects were sampled in aerated niches, but some species were found in watertight coffins. Therefore, the integrity of the closure and coffin affects colonisation. Sampled insects appeared dry, and not all identifications were clear. Molecular identification confirmed morphological identification. This result is interesting because older samples were estimated at 4 years old in these burial conditions. Previous studies were conducted on samples stored at room temperature [26]. Two Diptera species, *Hydrotaea capensis* and *Megaselia scalaris*, were the most abundant, supporting the hypothesis that insects can colonise carcasses in aerated burial systems. These two former species are much smaller than blow flies and flesh fly larvae, allowing them to manoeuvre through interstitial spaces to reach a carcass [27]. One year after the start of the trial, the coffins used for aerated burials had cracks, likely due to the decomposition activity of the carcasses. Perhaps this facilitated colonisation by *M. scalaris* larvae. This species tolerates darkness, and its ability to dig was experimentally proven in re-exhumed corpses buried in wooden coffins at soil depths of 30 and 60 cm. It can produce substantially large populations in the absence of other insects that arrive in the earlier stages of decay [26]. Our research confirmed the affinity of *H. capensis* for colonising buried corpses as the second most abundant species in our study. This finding is consistent with what is known for different species of the genus *Hydrotaea* sp. and for *H. aenescens* [28].

*Fannia pusio* provided relevant data. *Fannia pusio* (Wiedemann) is originally from the tropical and subtropical regions of the Americas, where it has been repeatedly collected from animal carcasses and human cadavers. This species is expanding its presence area and has been introduced to Africa, Asia, Australia, Oceania, and Europe [29]. However, further investigation of these data is needed.

We observed contamination in coffins that were opened post-removal. Most of the collected insects were necrophagous, capable of feeding on completely dry tissues. Others were omnivorous and opportunistic and, therefore, capable of colonising the bodies at any time of decay. In addition, we observed specimens of stored-product pests attracted by wood, textiles, and the remains of pioneer insects associated with the skeletons [30].

For example, the necrophagous moth *Monopis imella*, the beetle *Dermestes maculatus*, and the omnivorous and opportunistic feeders *Harpalus* sp. indicate surface decomposition and do not colonise buried bodies. For this reason, they are included in the post-removal from a niche context. Other coleopterans sampled included the carpet beetle *A. verbasci*, which feeds on textiles [30].

Although some points of criticism may be made in our study, such as partially recording temperatures within the different niches, this paper could offer first-hand data on faunal diversity in burial contexts and provide valuable information in forensic cases in many countries.

## 5. Conclusions

Our research presented two key results:

i. In bodies buried in a watertight environment (where a coffin is placed inside a sealed niche), skeletonisation occurs much more slowly with respect to bodies entombed in the same environment but aerated. It is probable that bacteria rapidly consume the oxygen inside the sealed metallic coffin. This creates a micro-environment that promotes an almost indefinite preservation of the body.
ii. The rapid decomposition of bodies under aerated conditions, probably due to increased circulation of air and entomofauna, valuable elements in the degradation of organic matter.
iii. The body concealment in coffins significantly affected the composition of the cadaveric fauna. The results reported in this paper emphasise how the burial system plays a key role in the selection of entomofauna associated with body colonisation. Our data, in agreement with previous work [31], highlight the role of *M. scalaris* and *H. capensis* in the colonisation and recycling of organic material in bodies located under confined conditions, unlike in exposed bodies, where blowflies are the most important taxon in body colonisation.

The aerated burial system could therefore represent a new, greener option. Furthermore, these burials allow plenty of ventilation, which enables a more hygienic and faster decomposition of bodies compared to watertight entombment, i.e., the most traditional techniques in Italy.

## Figures and Tables

**Table 1:** Time of exhumation details for each burial niche.

Type of Niche	Identification Niche	Time of Exhumation (Month)
Aerated	AX	6 and 60
	A1	12, 36 and 60
	A2	24 and 60
	A5	60
Watertight	Z1	12, 36 and 60
	Z2	24 and 60
	Z5	60

**Table 2:** Average daily air temperature at 2 m above ground (°C).

Month	2016	2017	2018	2019	2020	2021
January		2.1 ± 1.7	6.3 ± 2.1	2.9 ± 1.9	4.7 ± 2.2	3.5 ± 1.9
February		7.3 ± 1.5	4.2 ± 2.7	7.2 ± 2.1	8.7 ± 1.5	7.8 ± 3.2
March		12.4 ± 1.9	7.6 ± 4.1	11.3 ± 2.2	9.9 ± 2.4	9.8 ± 2.1
April		14.7 ± 2.4	16.6 ± 3.2	13.8 ± 2.2	15 ± 2.9	12.3 ± 3.3
May		19 ± 3.9	20 ± 2.9	15.3 ± 2.9	19.2 ± 1.7	17.2 ± 1.5
June		24.9 ± 2.1	23.7 ± 2	25.8 ± 2.3	22.4 ± 2.9	24.7 ± 2.4
July		25.9 ± 1.8	26 ± 2	26. ± 2.7	25 ± 2.3	25.8 ± 1.7
August		26.6 ± 2.7	26.2 ± 2.8	25.9 ± 1.4	25.6 ± 2.5	25 ± 3
September		18.5 ± 2.3	21.7 ± 2.8	20.7 ± 2.8	21.5 ± 3.4	21.4 ± 1.9
October		15.3 ± 1.6	16.6 ± 1.8	16.8 ± 2.1	14.3 ± 2.5	14.5 ± 2.5
November	10.3 ± 3.9	8.7 ± 2.6	11.1 ± 3.7	10.6 ± 1.8	9.5 ± 3.4	10.8 ± 1.5
December	3.8 ± 2.4	3.9 ± 1.6	3.5 ± 2.7	6.2 ± 2.7	5.8 ± 2.3	

**Table 3:** The sum of cumulative daily precipitation (mm/day). Nd= No data.

Month	2016	2017	2018	2019	2020	2021
January		7.4	6.4	31.2	26.4	33.6
February		61	111.6	15	2.6	5.8
March		9.8	62.8	6.4	20.4	0.2
April		21.2	13.2	40	17.8	0
May		69.2	62.8		20.6	Nd
June		18	50.8		43.4	Nd
July		4.8	19.2	43.2	111.6	Nd
August		5.4	33.2	12.2	75	Nd
September		137.4	47.8	75	36.6	Nd
October		49.6	63.8	41.2	67.6	Nd
November	46.4	94.4	76.6	160	12.8	Nd
December	10	12.2	15.2	64.8	77.6	

**Table 4:** Timing of taxon collected from pig carcasses placed in Aerated and Watertight niches.

Taxon	12 Months	24 Months	36 Months	60 Months
Diptera				
*Megaselia scalaris* (Loew, 1866) (Phoridae)	Aerated	Aerated	Aerated + Watertight	Aerated + Watertight
*Hydrotaea capensis* (Wiedemann, 1818) (Muscidae)			Aerated + Watertight	Aerated + Watertight
Drosophilidae				Aerated
*Fannia pusio* (Wiedemann, 1830) (Fannidae)				Aerated
Heleomyzidae				Aerated
Coleoptera				
*Atheta* sp. (Staphylinidae)				Aerated
*Otiorhyncus* sp. (Curculionidae)				Aerated
Corylophidae				Aerated
Lepidoptera				
*Monopis imella* (Hübner, 1813) (Tineidae)				Aerated + Watertight
Psocodea				
Liposcelididae			Aerated	Aerated
Araneae				
Gnaphosidae			Watertight	
Isopoda				
*Porcellio pumicatus* (Budde-Lund, 1885) (Porcellionidae)				Watertight

**Table 5:** Comparison between morphological and molecular identification. * COI region amplified and sequenced (+), COI region NOT amplified (−).

Type	Morphological Identification	COI	Molecular Identification
Aerated	*Fannia* sp. (Diptera: Fannidae)	+	100% identity *Fannia pusio*
Aerated	*Hydrotea* sp.(adult) (Diptera: Muscidae)	+	100% identity *Hydrotaea capensis*
Aerated	*Hydrotea* sp.(pupa) Diptera: Muscidae)	+	mixed sequence
Aerated	*Megaselia* sp.(adult) (Diptera: Phoridae)	+	100% identity *Megaselia scalaris*
Aerated	*Megaselia* sp.(pupa) (Diptera: Phoridae)	+	99.19% identity *Megaselia scalaris*
Aerated	*Monopis* sp. (Lepidoptera: Tineidae)	−	
Aerated	*Hydrotea* sp.(pupa) (Diptera: Muscidae)	+	mixed sequence
Aerated	*Monopis* sp. (Lepidoptera: Tineidae)	−	
Watertight	*Megaselia* sp. (empty puparium) (Diptera: Phoridae)	+	100% identity *Megaselia scalaris*
Watertight	*Anthrenus* sp. (empty puparium) (Coleoptera: Dermestidae)	−	
Watertight	*Anthrenus* sp. (empty puparium) (Coleoptera: Dermestidae	+	mixed sequence

**Table 6:** Presence of bacterial microflora in different burial systems.

Burial Type	Presence of Bacteria	Anatomical Regions
Watertight (Z1)	*Vagococcus fluvialis * *Bacillus simplex * *Providencia stuartii * *Proteus* sp.	Hind limb
Watertight (Z2)	Negative	
Watertight (Z5)	Negative	
Aerated (AX)	*Bacillus licheniformis*	Hypostatic side
Aerated (A1)	*Bacillus licheniformis * *Bacillus subtilis * *Staphylococcus epidermidis*	Mouth Abdominal skin Hind limb
Aerated (A2)	*Bacillus pumilus * *Virgibacillus proomii*	Abdominal skin Hind limb
Aerated (A5)	*Virgibacillus proomii * *Bacillus licheniformis*, *Acinetobacter ursingii * *Aliciclobacillus acidoterrestris*	Mouth Head Throat Chest Abdomen Hind limb

## Data Availability

Data sharing is not applicable.

## Supplementary Materials

The following supporting information can be downloaded at: https://www.mdpi.com/article/10.3390/insects14060566/s1, Supplementary File S1: Glossary; Table S1: Insect sampling in different burial systems; Table S2: COI sequences of insects obtained.
